# Concomitant radiofrequency Maze III – procedure and surgical ASD closure in adults

**DOI:** 10.1186/1749-8090-8-S1-P53

**Published:** 2013-09-11

**Authors:** OS Golovenko, VV Lazoryshynets, VP Zalevskiy, BB Kravchuk, VV Sakalov, IA Perepeka, MM Rudenko, KV Rudenko, OZ Paratsiy, YP Truba

**Affiliations:** 1 Department of Congenital Heart Defects Surgery, Amosov National Institute of Cardiovascular Surgery, Kyiv, Ukraine; 2 Department of Electrophysiology, Amosov National Institute of Cardiovascular Surgery, Kyiv, Ukraine; 3 Department of Myocardial Pathology, Amosov National Institute of Cardiovascular Surgery, Kyiv, Ukraine

## Background

Atrial fibrillation (AF) and others supraventricular arrhythmias are one of the most frequent preoperatively complications in adult patients with ASD. We present our experiences of surgical treatment of AF with ASD in adults.

## Methods

84 adult patients (mean age 40,8 ± 7,2 years) with ASD and different forms of AF were operated between 2005-2012 with next forms of AF: paroxysmal – 48, persistent – 26, long persistent – 10 patients. We performed radiofrequency “maze III” procedure and then ASD closure in CPB. Mean CPB-time was 64±18 min. Lines of ablation were performed point-by-point applications with exposition of 15 seconds. All patients were on amyodaron-therapy for the first three months. The follow-up period was from 24 to 36 months. Standard 12-lead ECG was done daily during the postoperative hospital stay and at 1, 3, 6, and 12 months postoperatively. To evaluate cardiac function and the recovery of the atrial contraction TTE was performed before discharge and at 3, 6, and 12 months postoperatively.

## Results

At 6 months follow-up sinus or atrial rhythm was present in 73 (86.9%) patients. 53(63,1%) patients have no need of antiarrhythmic therapy. Right atrial contractility was restored in 100% (84/84) and left atrial contractility in 67.9% (57/84) of patients. LVEF was increased from 0.43±0.02 to 0.52±0.10 (P <0.05). In 3 cases (3.6%) sinus node dysfunction was developed and needed permanent cardiac pacing (AAIR mode).

## Conclusions

The main hemodynamic factor in patients after “Maze III” procedure is preservation of AV-synchrony. The maintaining sinus rhythm and AV-synchrony is more important, than restoration of atrial systolic function. The mean LVEF increased in postop period. Independent risk factors for late AF recurrence were longer duration of AF (>60 months) (OR=2.721, p=0.025), increased left atrial size (OR=1.105, p=0.004). Duration of AF is predisposable factor for sinus node dysfunction development.

